# *Sanguinaria canadensis:* Traditional Medicine, Phytochemical Composition, Biological Activities and Current Uses

**DOI:** 10.3390/ijms17091414

**Published:** 2016-08-27

**Authors:** Andrew Croaker, Graham J. King, John H. Pyne, Shailendra Anoopkumar-Dukie, Lei Liu

**Affiliations:** 1 Southern Cross Plant Science, Southern Cross University, Lismore, NSW 2480, Australia; graham.king@scu.edu.au; 2 School of Medicine, The University of Queensland, St. Lucia, QLD 4072, Australia; j.pyne@uq.edu.au; 3 School of Pharmacy, Griffith University, Gold Coast Campus, Gold Coast, QLD 4222, Australia; s.dukie@griffith.edu.au

**Keywords:** black salve, sanguinarine, *Sanguinaria canadensis*, bloodroot, herbal, escharotic, skin cancer, alkaloid

## Abstract

*Sanguinaria canadensis*, also known as bloodroot, is a traditional medicine used by Native Americans to treat a diverse range of clinical conditions. The plants rhizome contains several alkaloids that individually target multiple molecular processes. These bioactive compounds, mechanistically correlate with the plant’s history of ethnobotanical use. Despite their identification over 50 years ago, the alkaloids of *S. canadensis* have not been developed into successful therapeutic agents. Instead, they have been associated with clinical toxicities ranging from mouthwash induced leukoplakia to cancer salve necrosis and treatment failure. This review explores the historical use of *S. canadensis*, the molecular actions of the benzophenanthridine and protopin alkaloids it contains, and explores natural alkaloid variation as a possible rationale for the inconsistent efficacy and toxicities encountered by *S.* *canadensis* therapies. Current veterinary and medicinal uses of the plant are studied with an assessment of obstacles to the pharmaceutical development of *S. canadensis* alkaloid based therapeutics.

## 1. Introduction

A number of pharmaceutical compounds have been developed from natural products used as traditional medicines [1]. *Sanguinaria canadensis* is a herbaceous North American plant with a history of ethnobotanical use [2]. The rhizome of the plant, also known as bloodroot because of its red latex, contains a number of biologically active alkaloids that have been investigated as potential drug candidates [3]. Despite their elucidation over 50 years ago [4], and significant activity in a range of molecular pathways, the individual alkaloids of *S. canadensis* are yet to find utility as proven human therapeutics [5,6].

**Figure 1:**
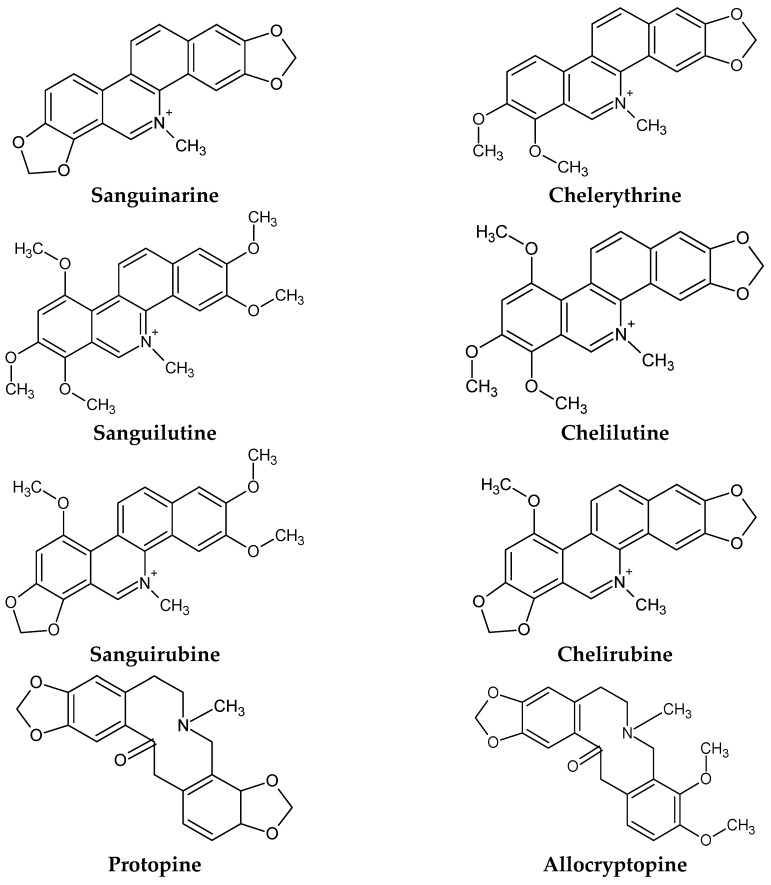
Chemical structures of biologically active *S. canadensis* alkaloids.

*S. canadensis* contains eight isoquinoline alkaloids at biologically relevant concentrations including six quaternary benzophenanthridine alkaloids (QBAs) sanguinarine, chelerythrine, sanguilutine, chelilutine, sanguirubine, chelirubine and two protopin alkaloids protopine and allocryptopine (Figure 1) [7]. Most reports regarding these alkaloids discuss their anticancer effects, especially for sanguinarine and chelerythrine [8], although antimicrobial [9], cardiovascular [10], neuroreceptor [11] and anti-inflammatory activities [12] of these alkaloids have also been identified.

With its promising biological activities, *S. canadensis* containing products have been used in vivo as livestock feed supplements [13], dental antibacterial agents [14], homeopathic arthritis [15] and skin cancer treatments [16]. The human use of *S. canadensis* containing therapeutics has been controversial. Mouth washes containing bloodroot alkaloids were in clinical use but subsequently found to induce leukoplakia and withdrawn from sale [17]. As a self-administered skin cancer cure, topical bloodroot therapies known as black salve have been associated with toxicity [18] and therapeutic failures [19].

Being based on a plant extract, the natural variation of biologically active alkaloids in *S. canadensis* may contribute to these unpredictable clinical effects. A number of factors including plant maturity, harvest season, growing environment and genetics have been found to influence the composition and concentration of these alkaloids [20]. Bloodroot harvested from separate geographic locations can exhibit significant alkaloid profile variation with individual rhizomes having an up to 15-fold difference in sanguinarine concentration [21]. Such botanical variation could inadvertently carry over to therapeutic products where the concentration of active compounds needs to be high enough to achieve desired clinical effects but not exceed levels likely to cause toxicity.

Understanding the molecular action of the phytochemicals in *S. canadensis* assists in identifying potential plant uses and mechanisms of toxicity. Previous reviews on bloodroot alkaloids have focused on the activity of individual compounds [22] or an aspect of their biological action [23]. As vintage drug leads, some having suspected normal cell cytotoxicity [24] and mutagenic potential [25], it is unclear whether the alkaloids from *S. canadensis* will be able to transition from bench to bedside. Drug discovery efforts have recently broadened to include multicomponent drug regimens acting in concert on multiple targets [26]. This review explores the clinically relevant alkaloids present in *S. canadensis*. Identifying the synergistic and antagonistic effects of bloodroot compounds may facilitate the development of a lower toxicity polypharmaceutical product.

## 2. Botany of *S. canadensis*

Correct identification and understanding of *S. canadensis* is critically important for the research and development of this herb into a therapeutic product. Bloodroot (*Sanguinaria canadensis* Linn.) is a flowering herbaceous perennial, native to eastern North America. Its range extends in a patchy distribution from Florida to Nova Scotia and west to the Rocky Mountains [27]. It is one of the earliest plants to flower in spring covering woodland areas with white flowers. Bloodroot is a member of the Papaveraceae, a family of lactiferous latex producing plants. Cutting the rhizome releases a red blood-like sap from which the plant derives its name.

The first documented botanical description of *S. canadensis* occurred in the work *Historia Plantarum Canadensium* by Cornuti in 1635, where the plant was named *Chelidonium americanum flore albo* [28]. Other names were proposed for the species with *Rannunculus virginiensis albus* suggested by Parkinson in 1640 [29] and *Papaver corniculatum* suggested by Plunkenet in 1696 [30]. It was the French florist Pierre Morin in 1651 who suggested the name *Sanguinaria*, derived from the Latin *sanguis* meaning blood in recognition of the rhizomes latex, and *canadensis* describing a region of the plants range [31].

Johann Jacob Dillenius, a Professor of Botany at Oxford University, first recognized the plant was a unique genus [32], while Linnaeus is credited with defining the genus in his book “*Species* *Plantarum*” where he reduced several Dillenius species to one under the name *Sanguinaria canadensis* [33]. Rafenesque subsequently identified several varieties Parviflora, Cespitosa, Reniformis, Repens, Multipetala (double petals) and Stenopetala (narrow linear petals) [34].

Bloodroot flowers are hermaphroditic being able to self-pollinate or cross-pollinate. Self-pollination cannot occur during the initial female phase as the stamens are positioned to avoid stigma contact. During this 1–3 day period, bees are the main pollinators. If pollination has not occurred after this time, the stamens bend down contacting the stigma which results in self-pollination [35]. Flowering, in the Northern hemisphere, lasts a few days and occurs from March until May with fruiting in June.

Bloodroot seed pods develop from May through early July. The seeds they contain are round, shiny red and half surrounded by a white vermiform raphe. Approximately 50 seeds are released from each oblong pod through two uplifting valves [36].

*S. canadensis* is a myrmecochore relying on seed dispersal by ants [37]. Myrmecochorous plants are distributed throughout the world but are concentrated in Australia (1500 species) and South Africa (1300 species) with only 300 species found in other regions of the world [38]. The lipid rich elaiosome is a food source that attracts ants which transport the seeds back to their nest. Seeds are discarded after the elaisome is eaten, *S. canadensis* germination rates have been found to increase after elaiosome removal by ants [39]. This method of propagation may explain the patchy clumping of *S. canadensis* throughout its distribution and the genetic relatedness of local populations [37]. Introduced fire ants (*Solenopsis invicta*) pose a threat to bloodroot seed distribution. They significantly decrease native ant species [40] and in eating the elaisome they often damage the seed, depositing it in areas unsuited to germination. As the range of fire ants has extended, so has the threat to bloodroot [41,42].

After the flower has dehisced, the leaf reaches its full expansion, the plant having a height of 15–30 cm. The leaf is palmate with seven to nine lobes and persists until August. Its upper surface is light green, while the undersurface is reticulated with prominent orange colored veins and has a grey down covering [43]. By the middle of summer, the aerial part of *S. canadensis* dies to the ground. Dormant plants can tolerate temperatures to −20 °C and are considered hardy [44].

Bloodroot grows best in light-to-medium well-drained soils and can tolerate a wide pH range, with optimal growth in soils of pH 5–7 or higher [45]. Its favored position is open woods but it may also be found along fences and in tree fall clearings. *S. canadensis* grows more successfully in low C:N ratio soils as evidenced by increased leaf and flower numbers [46]. Conflicting reports exist on the effect of elevation on Bloodroot growth [46,47].

The rhizome of *S. canadensis* is reddish-brown externally, 2.5–10 cm in length and up to 4 cm in diameter. The rhizome is truncated with abrupt ends and covered with orange-red colored rootlets [43]. When cut, *S. canadensis* rhizomes exude a thick red latex that contains a number of alkaloids. These bioactive defense chemicals help to protect the plant from micro-organism, insect and herbivore predation [11].

## 3. Historical Uses of *S. canadensis*

Historical uses of herbs can provide valuable information for the research and development of new therapies. *S. canadensis* has been extensively used to treat a number of symptoms and conditions by Native Americans and European colonists [48]. Adverse effects however were also recognized, suggesting the herbs therapeutic or toxic action may be strictly dose-dependent.

### 3.1. Traditional Native American Uses of S. canadensis

Native American people, especially of the Algonquian, Iroquois and Siouan language groups, developed a range of uses for *S. canadensis* [49], which they called poughkone or puccoon. It was highly valued as a skin dye when mixed with walnut oil or bear’s grease [50] and was attributed with magical properties by Poncan bachelors, who used it as a love charm [51]. It was also used as a traditional medicine to treat a variety of symptoms and conditions.

Several tribes used bloodroot to treat colds and congestion as either a tea or an inhaled powder [52]. The Ojibwe of Wisconsin used bloodroot as a lozenge for sore throats by adding it to a cube of maple sugar [53]. It was also used to treat diphtheria [54] and cases of haemorrhagic tuberculosis [52] where a small piece of bloodroot was added to concoctions, as larger doses would cause poisoning.

*S. canadensis* also had gastrointestinal effects with some tribes using small doses to stop vomiting [55] while other tribes used larger doses as an emetic [56]. It was made into a tea with blue cohosh to treat abdominal cramps [57] and was used to treat gastrointestinal haemorrhages and abdominal lumps [52]. Two tribes used bloodroot to treat haemorrhoids with the Malechite Indians heating the rhizome in a small kettle which was used when steaming [58] and the Cherokee using a bloodroot dampened cloth for manual reduction [52].

Native American women used bloodroot when having menstrual difficulties [52] and also used it as an abortificant [59]. The elderly used it as a tea to help their rheumatism while the Rappahannocks used it to purge fever [60].

*S. canadensis* was extensively used as a topical therapy by many tribes. Rhizomes were boiled in 1–2 liters of water, the liquid cooled to body temperature and applied as a coagulant to axe wounds [52]. Rhizomes were also applied to wounds as a poultice, while the Meskwaki would chew the roots and apply the masticated spittle to relieve the pain of burns [61]. The antibacterial properties of bloodroot were known to Native Americans who used it to treat wound infections [62] and gangrene [58]. The dry powdered rhizome was also used as an escharotic [63].

### 3.2. Early Western Use of S. canadensis

European colonists, through their interaction with Native American peoples, learned of the medicinal value of *S. canadensis*. Medical practitioners began using bloodroot for the treatment of Western illnesses [64]. During their investigations, the rhizome was confirmed as being of medical benefit while the leaves and seeds of the plant induced tremors, headache and torpor described as a stupefying or narcotic effect [65].

Respiratory uses of bloodroot rhizomes include as a bronchial muscle relaxant for asthma [66], a treatment for croup [67], influenza and whooping cough [68], an expectorant at a dose of one to two grains (one grain is equivalent to 64.5 mg) every second hour [43], an antibacterial agent to treat diphtheria and pneumonia [69], a treatment for tuberculosis [66] and as an inhaled powder to treat nasal polyps [70].

Bloodroot was also used to stimulate appetite [71], treat dysentery [72], functional dyspepsia [73], jaundice and chronic liver disease [74] and as a cure for alcoholism [75]. The administration of twenty grains (about 1.29 g) was used as an emetic [76]. It was also used as an emmenagogue to restore menses [77]. Bloodroot was also used to treat rheumatism suggesting anti-inflammatory properties [78].

During this period, an expanding range of topical applications was developed. A tincture made of bloodroot, rose water and vinegar was applied as a wash for eczema, ringworm and facial pimples. With the addition of glycerine it was used to rejuvenate hair follicles and prevent baldness [79]. A *Sanguinaria* nitrate ointment was employed for treating indolent ulcerative conditions of the skin, throat and anal fissures [79] while for syphilitic chancres it was used in a powdered form [80].

An American surgeon, Jesse Weldon Fell, learnt of the Cherokee Indians use of bloodroot to treat cancer, and found that zinc chloride enhanced its action [81]. Fell pioneered the combination of these two agents into a cancer salve first reported in the medical literature in the Lancet in 1857 [82]. Similar topical treatments known as Black Salve remain in use today.

## 4. Phytochemicals in *S. canadensis*

With its history of ethnobotanical use and apparent multi-system activity, *S. canadensis* has been studied to identify its active constituents as potential pharmaceutical agents. Most of this work has focused on the alkaloids contained within the rhizome, although long chain alcohols (C26–C34), phytosterols and triterpines have also been detected in the plant [83]. With bloodroot currently used for livestock and human therapeutic applications, it is important to understand the bioactive alkaloids it contains. As compound concentrations likely impact on product efficacy and toxicity, an understanding of factors that influence alkaloid levels in the plant is also of clinical relevance.

### 4.1. Identification of Alkaloids in S. canadensis

In 1827, Dana isolated a copper red extract from *S. canadensis* that was named sanguinarine [84]. Several years later in 1839 a different extract was isolated and named chelerythrine [85]. However, both these compounds were actually collections of alkaloids, with pure chelerythrine not being isolated until 1893 [86], and pure sanguinarine not being isolated until 1924 [87]. Sanguinarine and chelerythrine are the major quaternary benzophenanthridine alkaloids (major QBA) present in *S. canadensis*.

Following the development of column chromatography in the 1950s, a number of new benzophenanthridine alkaloids were discovered in *S. canadensis*. Being present in lower concentrations, they are termed the minor quaternary benzophenanthridine alkaloids (minor QBA). Chelirubine and chelilutine were first isolated from *Chelidonium majus* constituting 0.013% and 0.002% of its alkaloids (Slavik, 1954). They have also been obtained from a number of Papaveraceae species with *S. canadensis* rhizomes, to date, having the highest content of these minor QBAs at 0.074% and 0.229% respectively [4,88]. Sanguilutine and sanguirubine were isolated in 1960 from *S. canadensis* [4]. In an analysis of six plant species known for their alkaloid content, these minor alkaloids were only detected in *S. canadensis* [89].

### 4.2. Alkaloid Distribution in Different Plant Tissues of S. canadensis

**Figure 2:**
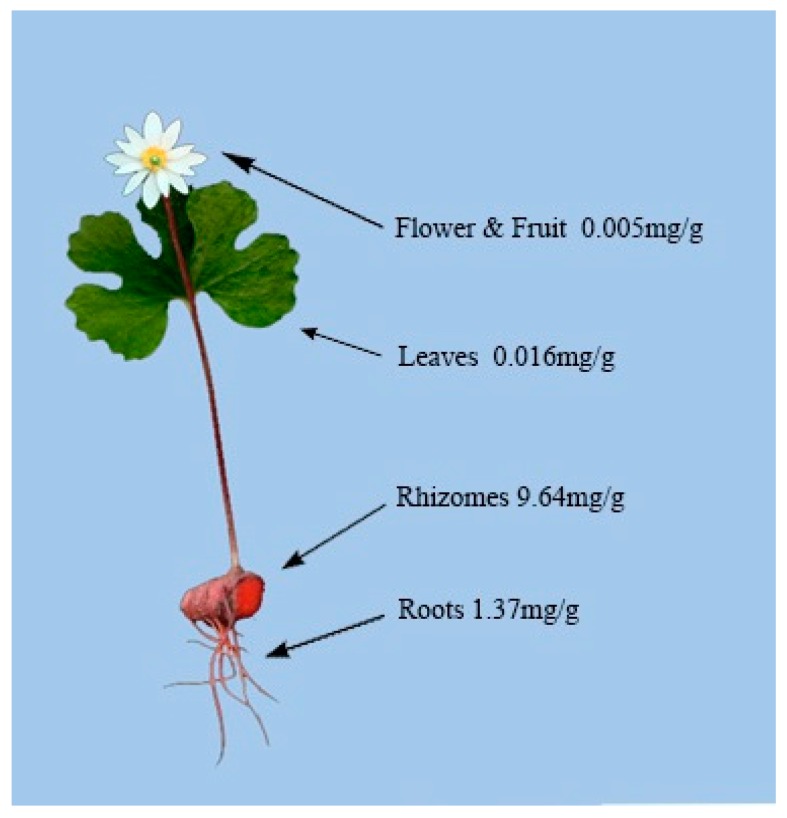
Average annual Sanguinarine concentrations in *S. canadensis* [90].

The distribution of sanguinarine in different *S. canadensis* tissues is shown in Figure 2 [90]. Sanguinarine has its greatest concentration in the plants rhizome, followed by the roots, with minimal concentrations present in the leaves and flowers. The distribution of other alkaloids within *S. canadensis* is currently not documented.

### 4.3. Rhizome Alkaloid Composition and Variation

The first exhaustive phytochemical study of *S. canadensis* rhizomes was performed by Slavik and Slavikova of Masaryk University Czechoslovakia in 1960 using a pseudocyanide isolation technique and column chromatography [4]. They imported undamaged rhizomes from a Chicago firm in the United States. *S. canadensis* rhizomes were found to contain an alkaloid pool consisting of the major QBAs sanguinarine (36.5%) and chelerythrine (33.4%), and the minor QBAs sanguilutine (9.1%), chelilutine (7.8%), chelirubine (2.5%) and sanguirubine (1.2%) [4]. In addition, the protopin alkaloids protopine (4.1%) and allocryptopine (4.2%) and trace amounts of the protoberberine alkaloids berberine and coptisine 0.1% were also identified [4].

Subsequent studies analyzing rhizome alkaloid composition in wild US populations have shown considerable natural alkaloid variation. The largest study assessed 100 *S. canadensis* populations over a range extending from North Carolina to Vermont, and west to Indiana. Plants were sampled when flowering, fruiting, post fruiting and prior to leaf senescence with 10–40 plants collected from each site [21]. Sanguinarine was found to contribute over 50% to the *S. canadensis* alkaloid pool with rhizome dry weight concentrations varying from 0.6% to 6.3% (average of 2.7%) [21]. Confirming earlier work [91,92], rhizome sanguinarine levels displayed a seasonal variation being maximal during flowering and early fruiting. The presence and concentration of alkaloids other than sanguinarine were not reported by the authors.

Cheleryrthrine, the other major QBA found in *S. canadensis*, also displays seasonal concentration variations in wild populations, being lowest during the Northern hemisphere summer months between July and September [93]. Comparing wild to cultivated bloodroot populations, rhizomes collected from May to November showed similar alkaloid concentrations. However, this collection time occurred outside the period of anthesis and early fruiting when wild alkaloid rhizome content is maximal.

Another study included the minor QBAs in its alkaloid rhizome analysis. Rhizomes were collected from 42 sites along an elevational transect between Athens, Georgia and Franklin, North Carolina. Bloodroot samples were collected when plants were in dormancy and at late seed set, both periods associated with low rhizome alkaloid concentrations. They found higher minor QBA and protopin alkaloid levels compared to those reported by Slavik. The alkaloid pool consisted of sanguinarine 30.4%, chelerythrine 18.8%, sanguilutine 12%, chelilutine 11.3%, chelirubine 9.5%, sanguirubine 0%, protopine 9.5% and allocryptopine 8.4% [20]. Lacking minor QBA standards, study data potentially relating to sanguirubine was excluded due to the coelution of sanguirubine and berberine high performance liquid chromatography (HPLC) peaks.

Several studies that assessed seasonal rhizome alkaloid variation in wild *S. canadensis* showed that the current understanding of *S. canadensis* alkaloid composition is incomplete [21,94,95]. This is especially the case for chelerythrine, the minor QBA and protopin alkaloids around anthesis when alkaloid concentrations are likely maximal [21,94,95].

Individual *S. canadensis* populations can also have significant alkaloid profile variations, with a 18.7 mg/g chelirubine yield (five times higher than the samples major QBA chelerythrine level) [94], or a 9% sanguinarine level, three times above the average rhizome content [21]. As bloodroot has been used as a base ingredient for livestock and human applications, levels of individual alkaloid exposure may be difficult to predict.

### 4.4. Other Factors Affecting S. canadensis Rhizome Alkaloid Concentrations

Both environmental and genetic factors may affect *S. canadensis* alkaloid production. The concentration of some bloodroot alkaloids, especially the majority of the benzophenanthridine alkaloids, appears to significantly depend on clonal genetics with an up to 10-fold concentration variance being attributed to clone. This genetic influence was not however universal, with chelirubine and protopin alkaloid concentrations found to be unrelated to clone [20].

Pressures on plant populations by pathogens and herbivores are known to increase with decreasing latitude [96,97]. Not surprisingly, there are increasing rates of alkaloid-bearing plants and an increase in the concentration of those alkaloids with decreasing latitude [98]. *S. canadensis* alkaloid concentrations were found to significantly reduce above 40 degrees latitude north of the Pennsylvania-West Virginia border [21].

Herbivory also often declines with increasing elevation reducing the need for costly resource consuming alkaloid chemical defenses [99,100]. Elevation also has an effect on *S. canadensis* alkaloid production with all QBA and protopin type alkaloid concentrations reducing with increasing elevation except for chelerythrine [47].

Cultivated rhizomes are often slightly larger than wild crafted bloodroot but have lower alkaloid content, perhaps due to a reduced need for chemical defenses. Cultivated rhizomes do not display seasonal alkaloid variation like their wild rhizome counterparts [93].

Plant maturity has been shown in *Macleaya macrocarpa*, a member of the Papaveraceae, to result in 3 to 14-fold higher rhizome minor QBA concentrations when comparing 12 year old plants to 2 year old plants [101]. *S. canadensis* requires 4–5 years of growth to reach a harvestable size, with older plants similarly being found to have higher alkaloid concentrations [94].

Soil properties such as the carbon to nitrogen ratio have also been shown to affect *S. canadensis* growth, although the impact on alkaloid production has yet to be assessed [46]. While high fertilizer exposure has been shown to reduce alkaloid concentrations [20]. This may be due to a shift from defense to growth [45], an artefact from increased alkaloid vacuole volume or reduced alkaloid production due to root nutrient burn.

Increasing shade has been shown to increase the benzophenanthridine and protopin alkaloid concentrations in *S. canadensis* [20]. Benzophenanthridine alkaloids are photoreactive toxins that could result in autotoxic events when in high ambient light environments [102]. Plants in reduced shade may therefore lower their alkaloid concentration to reduce the risk of autotoxicity.

## 5. Biological Activities of *S. canadensis* Alkaloids

Alkaloids, as a chemical class, have been successfully developed into valuable therapeutics, while a number are also recognized as potent toxins and poisons. Some alkaloids may act as a medicine or a poison depending on their dose of administration. An understanding of the molecular effects of *S. canadensis* and its bioactive molecules is necessary to fully assess its therapeutic potential and toxicity.

### 5.1. Anti-Cancer Effects of S. canadensis Alkaloids

#### 5.1.1. Sanguinarine

Sanguinarine (C_20_H_14_NO_4_) is the most studied alkaloid of *S. canadensis*, existing in either a charged iminium (pH 2–6) or uncharged alkanolamine (pH 6.5–9.0) form [103,104]. While both forms exist at physiological pH [105], the alkanolamine form is more lipophilic, having a greater penetration and cellular availability [106]. The iminium form is biologically active with spectroscopic studies revealing it to be the form that binds to nucleic acids. High concentrations of nucleic acids cause an equilibrium shift that transforms alkanolamine to the charged iminium form [107]. Sanguinarine is thus able to move into cells and accumulate in the nucleus and nucleic acid rich organelles, where it is activated to exert its cellular effects [108].

Sanguinarine interacts with DNA via intercalation, having a binding co-efficient comparable to the anthracycline agents daunorubicin and doxorubicin [109,110,111]. Its binding impairs DNA polymerase inducing DNA strand breaks and cell death [11,112]. It also prevents DNA strand break reconnection through depletion of nuclear topoisomerase II [113,114], this enzyme being the target of the chemotherapy drug etoposide [115].

Sanguinarine binds and caps telomeres inducing rapid apoptosis [116,117]. It also binds G-Quadruplex oncogenes c-Myc, KRAS and C-kit resulting in cell cycle termination [118,119,120]. In addition, it has been found to target the cellular cytoskeleton, inducing irreversible microtubule depolymerization [121] which inhibits cell proliferation inducing cell death [122].

Triple Helix H-DNA is present in c-Myc and Bcl-2 haematological malignancies [123,124] and is associated with poorer outcomes in colorectal cancer patients [125]. Sanguinarine binds to and impairs H-DNA [126,127]. Sanguinarine also inhibits B-DNA to Z-DNA transition altering DNA supercoiling [128,129]. This in combination with its binding to core histones results in altered chromatin structure and gene expression [130,131].

Through its direct genetic and epigenetic effects, sanguinarine has been shown to alter gene expression in HeLa cells [130]. Exposure to a non-cytotoxic 2 μM sanguinarine concentration resulted in the down regulation of 378 genes, mostly involved in metabolic pathways. A total of 348 genes involved in cell signaling, cell adhesion, extracellular matrix (ECM) receptor interaction and complement coagulation cascades were also upregulated at this concentration. When increased to a 5 μM concentration, a significant number of apoptosis genes such as DIABLO (direct inhibitor of apoptosis binding protein with low isoelectric point) were activated, with 225 genes being upregulated and only 35 genes being downregulated [130].

Sanguinarine also interacts with a variety of RNA molecules. It strongly intercalates with messenger RNA (mRNA) poly (A) inducing self-structure formation disrupting poly (A) polymerase (PAP) transcription [132]. Each molecule of sanguinarine binds six transfer RNA (tRNA) base pairs by intercalation further interfering with protein synthesis. Sanguinarine also strongly binds double stranded RNA (dsRNA) that are involved in gene silencing and epigenetic regulation [133].

Sanguinarine additionally impacts on protein transcription as shown by the proteomic analysis of BxPC-3 pancreatic cancer cells when exposed to a 1 μM concentration. Sanguinarine altered protein expression >1.5-fold in approximately 5% of 3107 identified proteins. A significant proportion of proteins affected by sanguinarine were involved in cellular metabolism with 61 proteins being upregulated and 87 downregulated [134]. This confirmed sanguinarine’s pleotropic ability to effect a broad range of protein classes and target multiple critical cellular processes. Protein ubiquitination was the molecular pathway most affected by sanguinarine [134].

Apart from its direct action on cellular nucleic acids, sanguinarine exerts a cytotoxic effect in multiple cell lines via significant reactive oxygen species (ROS) generation [135]. Through redox cycling, apoptosis initiated by sanguinarine is p53 independent [136]. Conflicting results, however, regarding the role of ROS in sanguinarine’s cytotoxic action have been reported. Some studies suggest ROS play a critical role while others have shown sanguinarine to inhibit oxidative bursts through a direct action on NADPH (Nicotinamide Adenine Dinucleotide Phosphate) oxidase rather than redox activity [137,138]. Further work is needed to clarify these conflicting actions.

When assessing sanguinarine’s ROS anti-cancer effects in the SPCA1 human lung adenocarcinoma cell line, sanguinarine through ROS was found to induce endoplasmic reticulum (ER) stress [139]. ROS cause the unfolding and or misfolding of proteins that accumulate in the lumen of the ER [140]. This triggers a molecular cascade termed the unfolded protein response (UPR) [141]. Continuous ER stress promotes ROS formation creating a positive cytotoxic feedback loop [142]. For this reason, the UPR has been identified as an anti-cancer target [143].

Sanguinarine also causes a rapid and severe reduction in glutathione (GSH) levels. Although glutathione is an antioxidant, this depletion was due to a direct chemical action on GSH by sanguinarine, with 50% of cellular GSH being degraded within 1 min of exposure to a 5 μM sanguinarine concentration [24]. Such significant GSH depletions can be apoptogenic in their own right [144,145]. This reduction in cellular antioxidant reserves accentuates the damage from sanguinarine induced ROS.

**Figure 3:**
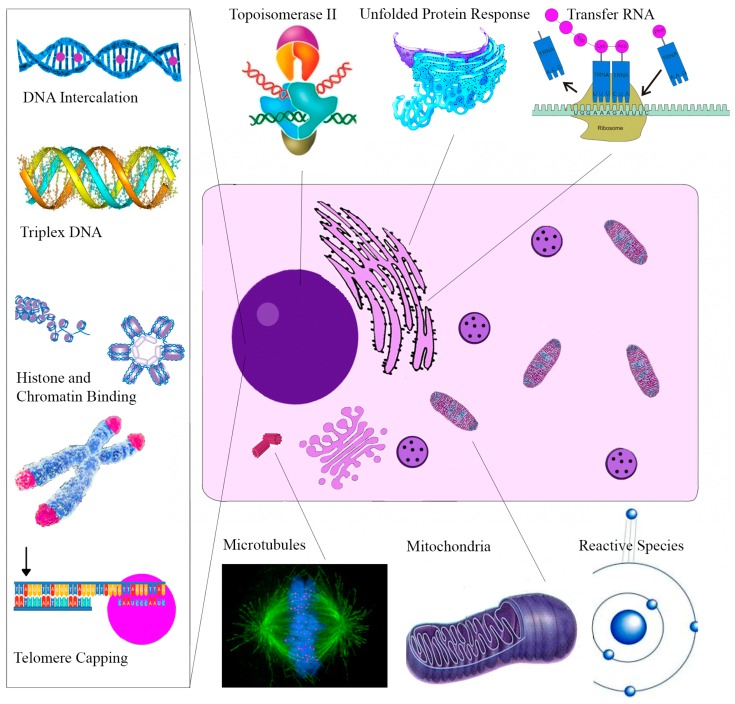
The cellular targets of sanguinarine. This figure highlights the variety of cellular organelles and molecular processes disrupted by sanguinarine. The only sanguinarine molecules (purple dots) in the diagram are represented in the DNA Intercalation image.

Tumor growth facilitated by neovascularization is another area targeted by sanguinarine [146,147]. At nanomolar concentrations, it inhibits vascular endothelial growth factor A (VEGF-A) induced endothelial cell migration, sprouting and survival [148,149]. Sanguinarine targets a range of cellular structures (Figure 3) and molecular processes. The contribution of each mechanism to the alkaloid’s cytotoxicity and anticancer action is not clearly understood.

#### 5.1.2. Chelerythrine

Chelerythrine is the second most prevalent alkaloid in *S. canadensis* having two methoxyl substituents at the C11 position on the aromatic D ring. Chelerythrine is less planar than sanguinarine and has a lower affinity for DNA [109]. Despite this, chelerythrine displays activity against a range of human tumors [150] including those that are radiotherapy, chemotherapy resistant [151] as well as p53-deficient [152,153].

Traditional high throughput drug discovery programs have identified chelerythrine as a lead oncology drug candidate. Of 107,243 extracts derived from plants, actinomycetes, fungi, marine invertebrates and marine bacteria, screened for their Bcl-XL and BH3 disrupting ability, 12 extracts had significant activity. Chelerythrine was the causative bioactive molecule in the four plant extracts with an (IC_50_ of 1.5 μM) [153]. A further study that screened 6700 compounds for mammalian target of rapamycin (mTOR) inhibitors found chelerythrine had the greatest cytocidal action against cells with mTOR hyperactivity [154].

#### 5.1.3. Minor Quaternary Benzophenanthridine Alkaloids (QBAs)

Less is known about the biological activities of the minor QBAs, which are generally present in lower concentrations in *S. canadensis*. Sanguilutine has greater cytotoxicity than the major QBAs with lower 50% inhibitory concentrations (IC_50_) [155,156]. With IC_50_ values ranging from 0.04 to 1.58 μg/mL, the minor QBAs are active at therapeutically relevant doses (Table 1).

The minor QBAs appear to act through molecular processes that differ from the major QBAs. Chelilutine acts as a strong inducer of apoptosis without ROS generation [156], while sanguilutine induces melanoma cell death via necroptosis [158]. Necroptosis is a novel non-apoptotic caspase-independent programmed cell death facilitated by Receptor-interacting protein kinase 1 (RIP1) [159]. Sanguilutine activates RIP1 resulting in Ripoptosome (RIP1, FADD and caspase-8) formation leading to cell death [158]. Cancer cells are often resistant to therapies due to altered apoptotic machinery. Compounds like sanguilutine that act by alternate pathways are of growing interest.

#### 5.1.4. Protopin Alkaloids

Unlike the benzophenanthridines, protopin alkaloids have minimal antiproliferative effects on a range of cancer cell lines (Sun et al., 2014). Protopine alleviates oxidative stress and apoptosis, partially by antioxidant mechanisms and Ca^2+^ antagonism [160]. Protopine may therefore antagonize the cytotoxic action of other *S. canadensis* alkaloids. Protopine has been found to impair the cancer cell invasion and metastatic potential of MDA-MB-231 breast cancer cells by reducing the expression of adhesion molecules such as epidermal growth factor receptor (EGFR), ICAM-1 and integrins [161].

Although having a similar chemical structure, the alkaloids present in *S. canadensis* have many unique molecular specificities (Table 2). Sanguinarine, for example, is a potent inhibitor of NF-κB, while chelerythrine lacks NF-κB activity [162]. When bloodroot alkaloids share a molecular target, they may bind different sites on the molecule, as is the case with the pro-survival protein Bcl-XL [163]. The impact of simultaneous target exposure to different alkaloids has not been extensively studied. Whether alkaloids would antagonize or augment target disruption is an unresolved question of direct clinical relevance.

### 5.2. Cardiovascular Effects of S. canadensis Alkaloids

Sanguinarine has been found to have a vasodilatory effect, inhibiting α-1 and α-2 adrenoceptors with an IC_50_ of 33.6 and 6.4 μM, respectively [176]. When investigating 19 Panamanian plants used as traditional medicines to treat hypertension, the extract of *Bocconia frutescens* L. containing sanguinarine [177], inhibited angiotensin II binding by 50% [178]. Sanguinarine has been shown to block angiotensin II in a slow, nearly irreversible and non-competitive manner [179]. In addition, protopine has vasodilator effects by elevating cAMP and cGMP [180].

Several bloodroot alkaloids interact with cardiac ion channels. Sanguinarine has been found to inhibit Na^+^-K^+^ ATPase activity, similar to the pharmaceutical digoxin, with an IC_50_ of 6.0–6.5 μM. In guinea pig atria this elevates intracellular Na^+^, activating the Na^+^/Ca^2+^ exchanger resulting in increased intracellular Ca^2+^ levels, a 10 μM dose having a positive inotropic effect [181]. Protopine acts as a promiscuous cation channel inhibitor in guinea pigs blocking Ca^2+^, K^+^ and Na^+^ ion channels [182], while allocryptopine blocks K^+^ channels in rabbit ventricular myocytes [183]. Allocryptopine is more effective than quinidine in preventing induced arrhythmias in rats [184]. Chelerythrine in cultured neonatal rat cardiac myocytes induces apoptotic death at a concentration of 6–30 μM due to the generation of ROS [185].

### 5.3. Anti-Inflammatory Actions of S. canadensis Alkaloids

Inflammation is induced by pro-inflammatory enzymes such as COX-2, NO and PGE2 and the inflammatory cytokines TNF-α, Interleukin IL1 and IL6. Protopine exerts an anti-inflammatory action being a potent NO inhibitor, reduces COX-2 expression and impairs the production of PGE2, IL-1B, IL-6 and TNF-α. This occurs through the inhibition of ERK 1/2 and JNK phosphorylation and NF-κB activation in murine macrophages [186]. Protopine has been found to inhibit carrageenan-induced rat paw oedema with a potency three-fold higher than acetylsalicylic acid [12].

Chelerythrine has also been found to exert an anti-inflammatory action by inhibiting COX-2 and PGE2 production [187]. The inhibitory COX-2 potency of chelerythrine is comparable to NS-398 a specific Cox-2 inhibitor. Chelerythrine also has an effect on 5-lipoxygenase inhibition [188] and oxidative burst attenuation [189,190].

Polymorphonuclear leukocytes (PMN) release cytokines and lytic enzymes that fuel inflammation, however, when PMN undergo apoptosis, they are removed by the reticuloendothelial system without inflammatory mediator release [191]. PMN apoptosis has been found to be a major mechanism of inflammation resolution in the gut, lung, joint and kidney [192,193,194]. Chelerythrine induces rapid apoptosis in human PMN via a PKC (Protein Kinase C) independent mechanism preceded by rapid caspase-3 activation. 10 μM chelerythrine chloride induces rapid and significant PMN apoptosis in less than 4 h [195].

Intercellular adhesion molecules (ICAM) and vascular cell adhesion molecules (VCAM) facilitate the migration of neutrophils into tissues [196] and are key components of inflammatory disease processes such as asthma [197,198] and inflammatory arthritis [199,200]. Testing of 40 natural and synthetic alkaloids, terpenoids and phenolics found sanguinarine and isoliquiritigenin significantly downregulated VCAM-1. Of the 10 compounds that downregulated ICAM-1, sanguinarine had the greatest effect, reducing expression by 50.6% [201].

### 5.4. Anti-Infectious Effects of S. canadensis Alkaloids

As a group, the benzophenanthridine and protopin alkaloids of *S. canadensis* have significant antimicrobial action. Sanguinarine has been found to induce the release of bacterial membrane-bound cell wall autolytic enzymes resulting in activity against Methicillin-Resistant *Staphylococcus Aureus* (MRSA) with a minimum inhibitory concentration (MIC) for two reference strains of 1.56 μg/mL and 3.12 μg/mL [202]. Sanguinarine has bactericidal activity against gram negative and gram positive vancomycin resistant organisms, when co-administered sanguinarine rendered the organisms vancomycin sensitive [203]. When tested against 64 different oral microbial species, sanguinarine’s MIC ranged from 1 to 16 μg/mL for 98% of isolates [204].

*Helicobacter pylori* has a pathogenic role in gastritis and peptic ulcer disease with increasing resistance rates to current combination antibiotic therapies [205]. A methanol extract of *S. canadensis* rhizomes had an MIC_50_ against *H. pylori* of 12.5 μg/mL while individual alkaloids were less potent with MIC_50_ values of 50 μg/mL for sanguinarine and 100 μg/mL for chelerythrine and protopine [206], suggesting the alkaloids may have a synergistic effect.

On testing 43 plant species traditionally used for treating tuberculosis, extracts of *S. canadensis* were shown to have antimycobacterial activity. On analyzing the activity of individual constituents, chelerythrine was found to be the most active with an IC_50_ of 7.3 μg/mL (19.02 μM) against *Mycobacterium aurum* with sanguinarine being the second most active with an IC_50_ of 9.61 μg/mL [207].

The alkaloids of *S. canadensis* also appear to have anthelmintic activity. Schistosomiasis is a snail-borne parasitic disease which affects over 200 million people worldwide, and is usually treated with Praziquantel [208]. New therapies are required as Praziquantel does not treat early infection (Magnussen, 2003) and schistosome resistance has been reported in endemic areas such as Egypt [209]. On testing 45 compounds, sanguinarine was one of two compounds where a 10 μM concentration resulted in 100% parasite mortality after 48 h, meeting the World Health Organisation’s compound “hit” criteria [210].

*Strongyloides stercoralis* is a parasitic infection affecting 100 million people in tropical zones worldwide [211]. Cure rates for current anthelmintics range from 84% for Ivermectin to 46% for Albendazole [212]. Protopine and allocryptopine have been demonstrated to show significant activity with average 50% larval paralysis concentrations of 42.5 and 66 μM, respectively (Satou, Koga, et al., 2002), with protopine’s ratio of parasite to human cell toxicity being superior to that of Ivermectin and Albendazole [213].

Parasitic infestation is an increasing issue in aquaculture, with freshwater fish at risk of gill monogenean infestation, especially Dactylogyrus [214]. Sanguinarine was found to have the greatest potency of *Macleaya microcarpa* (Maxim) Fedde aerial alkaloids with an EC_50_ of 0.37 mg/L. Less potent actions were recorded for 6-methoxyl-dihydro-chelerythrine 3.63 mg/L, allocryptopine 4.64 mg/L and protopine 8.13 mg/L [215].

Veterinary anthelmintic applications have been explored with alkaloids tested against dog roundworm *Toxocara canis*. Few anthelmintics are effective against nematodes and then often only when they are present in the gastrointestinal tract. Pyrantel pamoate has a relative mobility 50% (RM_50_) inhibition against intestinal *Toxocara canis* at a concentration of 46 μmol/L. Strong nematocidal activity has been observed for 6-methoxydihydrosanguinarine RM_50_ 18 μmol/L, chelerythrine RM_50_ 28 μmol/L and sanguinarine 58 μmol/L [216]. The authors argued that due to the HL60 cytotoxicity of these agents with IC_50_ values for 6-methoxydihydrosanguinarine of 0.3 μmol/L, chelerythrine 0.2 μmol/L and sanguinarine 0.5 μmol/L, other compounds would be better therapeutic candidates. However, the HL60 cell line chosen to determine alkaloid cellular toxicity, is more sensitive to alkaloid damage than non-malignant cells and may overestimate the potential toxicity risk [138]. Allocryptopine, had a RM_50_ of 349 μmol/L and an IC_50_ of 48 μmol/L, its lower RM_50_/IC_50_ resulted in its recommendation as a more promising compound for pharmaceutical development [216].

A screening study of 2000 drugs and natural products found sanguinarine had antiviral activity inhibiting HIV protease with an IC_50_ of 13 μM [217]. Sanguinarine also has activity against herpes simplex virus while protopine and allocryptopine are both active against Parainfluenza Virus-3 [218].

Protopine displays promising anti-plasmodial activity against TM4 wild type and multi-drug resistant (K1) *P. falciparum* strains with an IC_50_ value of 1.5 μg/mL [219]. Chelerythrine and sanguinarine were found to be especially active against the trypanosome *T. brucei* the cause of sleeping sickness with EC_50_ of 1.3 and 4.8 μM, respectively [220]. While the antifungal activity of protopine is uncertain, one study suggested minimal activity [221] while another showed strong inhibition of *Candida albicans* with a 4 μg/mL exposure [218].

### 5.5. Other Biological Activities of S. canadensis Alkaloids

There are also reports of the *S. canadensis* alkaloids having neurotransmitter, local anesthetic, gastrointestinal and coagulation effects. Plant alkaloids have provided acetylcholinesterase inhibitor drug leads for the treatment of Alzheimer’s disease; for example, galanthamine derived from *Galanthus woronowii* and Rivastigmine, a semi-synthetic derivative of physostigmine from *Physostigma venenosum,* with both in clinical use [222]. In vitro and in vivo studies have identified both protopine and allocryptopine as potent acetylcholinesterase inhibitors [223,224]. While galanthamine has a 49% inhibitory action for acetylcholinesterase, allocryptopine and protopine cause greater inhibition of 89.31% and 80.53%, respectively [225].

Chelerythrine has been investigated as a pharmacological agent to treat insulin resistance. In mice it more potently blocked peroxisome proliferator-activated receptor gamma (PPARγ) phosphorylation than thiazolidinedione (TZD) drugs. Its transcriptional profile suggested to have reduced side effects compared to existing TZD medications [226].

Protopine also has potential uses as a neuroprotective agent in stroke and as an antidepressant. Protopine has been shown to reduce brain glutamate levels by 23% by increasing glutamate dehydrogenase (GDH) 1.6-fold [227]. Glutamate excitotoxicity and calcium overload are involved in the pathophysiology of stroke. Protopine appears to increase superoxide dismutase activity in serum suggesting its neuroprotective effect is partly related to its anti-oxidant properties. Protopine has also been suggested as a treatment for mood disorders as it seems to inhibit both the serotonin transporter (SERT) and noradrenalin transporter (NERT) [228]. Sanguinarine blocks nicotinic and 5-HT2 receptors with an IC_50_ of 11.8 and 91.7 μM, respectively [176].

A 1% solution of raw alkaloids from *Macleaya cordata (*Wild.) that has allocryptopine as its major alkaloid constituent has a local anesthetic effect [229]. A 1% solution of these raw alkaloids has a stronger local anesthetic effect than procaine of similar concentration [230].

Allocryptopine appears to induce a relaxing effect on guinea-pig ileum via phosphodiesterase inhibition but has a contractile effect through its interaction with α-adrenoreceptors on the bladder [231]. Protopine is a weak anticholinergic alkaloid that is 660 times less potent than atropine but still possesses an anti-spasmodic and relaxant effect on smooth muscle [232]. Sanguinarine blocks muscarinic receptors with an IC_50_ of 2.4 μM [176].

Protopine also seems to act on platelets having a lower IC_50_ value against various platelet agonists such as arachidonic acid and platelet activating factor than those observed for aspirin [12]. While protopine selectively inhibits the synthesis of thromboxane A2, sanguinarine inhibits thromboxane B2 production [233].

## 6. Current Uses of *S. canadensis*

### 6.1. Agriculture and Aquaculture Feed Supplement

In 2006, the European Union ratified a ban prohibiting the use of antibiotics as animal growth supplements, due to increasing pathogen antibiotic resistance [234]. This has stimulated interest in phytogenic feed supplements that reduce gastrointestinal pathogen levels, enabling animals to redistribute immune defense resources to growth [235]. Thus, Sangrovit, derived from the aerial parts of *M. cordata* or the rhizomes of *S. canadensis*, was developed as a natural feed additive [236,237]. The sanguinarine content of Sangrovit was standardized to 1.5% *w*/*w*.

In a number of studies, Sangrovit has been shown to improve weight gain in weaning pigs [238], broilers [239] and tilapia [240]. Sangrovit significantly reduced *Salmonella enteritidis* positive crop and cecal samples in broilers 7 days post Sangrovit inoculation [241]. Pigs fed diets containing 1.5 g of QBAs/1000 kg of feed had more effective reduction in fecal *Salmonella* shedding than pigs administered chlortetracycline, a commonly used US feed antibiotic [242]. Sangrovit induced weight gain in fish appears due to appetite stimulation [240].

Due to toxicity concerns, Sangrovit 90 day feeding experiments at 50 times the recommended dose have been performed in rats and pigs. These did not show lymphocyte or hepatocyte genotoxic damage in rats [237], nor histological, haematological toxicity or DNA adduct formation in pigs despite plasma sanguinarine and chelerythrine levels reaching 0.11 and 0.2 μg/mL, respectively [13]. This suggests the ingestion of *S. canadensis* alkaloid supplements over a period of months is not associated with toxicity.

### 6.2. Veterinary Uses

Bloodroot-containing topical therapies have been used to treat equine sarcoids. While locally invasive, these common horse tumors rarely metastasize but often recur after treatment [243]. In veterinary medicine, current bloodroot formulations include XXTERRA (Larson Labs, Fort Collins, CO, USA), Newmarket bloodroot ointment (Newmarket Premixes, Catley Cross, Halstead, UK) and Animex (Nies, Las Vegas, NV, USA). The composition of Newmarket bloodroot is 40% *w*/*w* 1:3 extract (25% methyl alcohol/75% water) *S. canadensis* rhizome, 48% *w*/*w* emulsifying ointment, 10% zinc chloride and preservatives.

Small pilot studies have suggested equine sarcoid complete resolution rates comparable to Imiquimod [244,245]. With 57 owners reporting a 98% complete resolution rate for smaller sarcoids <2 cm diameter in a retrospective questionnaire [246]. Bloodroot products such as Neoplasene have also been used to treat tumors in dogs with a female Golden Retriever developing a fluctuant cutaneous mass following an intra-tumoral injection with subsequent post-operative complications [247].

### 6.3. Dental Antibacterial Treatment

The observed broad spectrum activity of *S. canadensis* alkaloids against oral micro-organisms led to the development of bloodroot based antiplaque and gingivitis therapies [204]. Viadent, a mouth rinse containing 0.3 mM sanguinarine chloride, and a toothpaste containing 0.7 mM sanguinarine chloride were offered as an alternative to chemical based oral antiseptics [248]. Viadent use, however, became associated with the development of leukoplakia, a pre-malignant condition, of the maxillary vestibule [249].

Although mouth rinsing is a brief activity of a few minutes duration, the maxillary vestibule has low salivary turnover and prolonged mucosal contact to rinse residue [250]. The safety of *S. canadensis* oral health care products had been assessed in a number of animal toxicity and human clinical efficacy studies [251], however, none of these studies were longer than 6 months’ duration. Of patients with maxillary vestibule leukoplakia, 84% had used Viadent over an average of 4.4 years use [252]. Cessation of Viadent did not result in leukoplakia regression, suggesting permanent epithelial cell genetic alteration [253]. While Viadent did reduce dental plaque levels, it was inferior to Chlorhexidine as a treatment for gingivitis and gingival bleeding [254] and was withdrawn from sale in North America due to concerns about its carcinogenic potential.

### 6.4. Homeopathic Treatment

Hylands menopause treatment has been sold in the United States as an over the counter therapy for hot flushes for over 50 years. A homeopathic medicine, it contains *S. canadensis* 3× (1:1000 dilution), amyl nitrate 3× (1:1000 dilution) and Lachesis, the poison from the bushmaster snake 12× (1:1,000,000,000,000 dilution) [255]. One randomized clinical trial with 83 subjects has been reported in the literature with a Jadad score of 5. Those using Hylands Menopause treatment had increased flush severity scores and developed headaches compared to the single compound remedy and placebo groups, suggesting a lack of efficacy for menopause [256].

An observational study of various homeopathic treatments in 438 patients with menopausal symptoms found *S. canadensis* remedies ranked fifth most likely out of 16 remedies to result in symptom disappearance or improvement. However, the study type and design prevented an assessment of efficacy [257].

Zeel comp. N (Zeel) is a homeopathic formulation derived from *Sanguinaria canadensis*, *Arnica montana* (Arnica), *Solanum dulcamara* (Climbing nightshade), *Rhus toxicodendron* (Poison Oak), and sulfur. Unlike classical homeopathic remedies, the therapy does not employ a single active ingredient and is not diluted “in extremis” having molar concentrations of 10^−5^–10^−12^ [258].

A number of studies have been conducted in patients with osteoarthritis. One single blinded study compared intra-articular Zeel given twice weekly with intra-articular sodium hyaluronate given once weekly over a five-week period [259]. Another double blind randomized study in 121 patients compared oral diclofenac 25 mg with oral Zeel, each tablet taken three times daily for 10 weeks [260]. The largest study, comparing Zeel tablets with COX-2 inhibitors, enrolled 592 patients in an open prospective multicenter reference controlled cohort study [261]. All of these studies reported equivalence between Zeel and conventional arthritis treatments.

### 6.5. Use in Cancer Therapy

#### 6.5.1. Mohs Paste

In 1932, Frederick E. Mohs, while investigating the reaction of cancerous and normal tissues to various irritants, observed that a 20% solution of zinc chloride chemically killed tissues yet maintained their histologic structure [262]. Mohs believed that skin cancer recurrence occurred as a result of tumor cells present at the surgical margin escaping detection by standard histology procedures that only inspect 1% of an excised lesion [263].

Mohs developed a fixative paste that would histologically preserve human tissue in a controlled manner. In addition to zinc chloride, he found that stibnite and an extract from *S. canadensis* rhizomes stabilized the paste [264], allowing it to function like a reservoir that slowly released zinc chloride into surrounding tissues [265]. Mohs fixed tissue technique involves a thin layer of paste fixing the tumor in-situ, with a horizontal section taken through the base of the lesion 24 h later. The entire excision margin is then histologically assessed with any tumor involvement being mapped. Further fixative is then applied to these areas which undergo additional tissue removal. The process continues until histologically clear margins are achieved [266].

The technique became known as Mohs micrographic surgery. Using the fixed tissue method, Mohs 5 year cure rate for basal cell carcinoma (BCC) in 7257 cases was 99.3% and for squamous cell carcinoma (SCC) in 2551 cases was 94.4%. This impressive result was achieved despite 20% of the patients having recurrent tumors following previous surgery or radiotherapy [267]. While *S.* *canadensis* is a component of Mohs paste, the high cure rates achieved by Mohs technique are attributable to the complete histological assessment of excision margins and not the anticancer action of *S. canadensis*, as demonstrated by the equivalent effectiveness of the later developed fresh tissue technique where margin controlled surgery is performed without a fixative paste [268].

#### 6.5.2. Salve Chemosurgery

In the 19th century, the American surgeon Jesse Weldon Fell learned that Native Americans living on the shores of Lake Superior used *S. canadensis* rhizomes to treat cancer. Through experimentation he found its effectiveness could be enhanced by the addition of zinc chloride [269]. These constituents were mixed in equal parts with water forming the treacle-like Fells Paste.

Fells Paste was trialed in twenty five patients at Middlesex Hospital London in 1857, the majority of cases being breast cancer [82]. Fell also applied a brown bloodroot containing ointment to treat lymph node metastases and an internal *S. canadensis* therapy containing half a grain three times daily [81]. The Middlesex surgeons concluded zinc chloride was the only active constituent in the paste with bloodroot having no anticancer activity [270].

Fell reported in the Lancet that of 453 cancer patients he treated with the paste, many of these being advanced cases not suitable for surgery, the recurrence rate was 27.5% at two years. He argued this was a significant advance on the 80% recurrence rate achieved by surgery at two years [81]. The independently observed results from the 25 patients at Middlesex Hospital showed a 9 months recurrence rate of 21.4%. This called into question the accuracy of Fells reported two year results. With 21.4% recurring by 9 months, it was not believed possible only 27.5% would recur by two years [270]. Unfortunately, two year recurrence rates for the Middlesex Hospital patients were not reported.

The provision of unproven bloodroot based oncology products to the public has not been an isolated event in medical history. In the early 20th century, another entrepreneur Harry Hoxsey [271], promoted a *S. canadensis* containing cancer therapy to the public. The Hoxsey cancer treatment utilized two external therapies, a yellow powder containing arsenic sulphide, sulphur, talc and yellow precipitate; also a red paste containing bloodroot, antimony trisulfide and zinc chloride [272]. Tonics were employed to treat internal malignancies but these did not contain *S. canadensis* [271]. There have been no clinical trials of the Hoxsey treatment. One observational study conducted from 1983 followed 39 cancer patients for 5 years. By the fifth year, 58.9% of patients had been lost to follow-up [273]. A pilot progressive cohort study of 149 patients, treated with the Hoxsey cancer treatment at the Tijuana Bio-Medical clinic, was conducted in 1992. Only 43.6% of records contained pathology reports with the 5 year vital status only assessable for 57% of patients. At 5 years, 11.4% of patients were alive, 45.6% had died and the outcome of 42.9% of patients was unknown [274]. These studies highlight the lack of scientific rigor employed by clinicians offering bloodroot oncology products and the unknown status of significant patient numbers, preventing an assessment of treatment efficacy.

More recently, black salve, a *S. canadensis* containing topical cancer therapy, has been sold to patients especially through online vendors [275]. Formulations vary with one of the more established treatments known as Cansema or Amazon by Alpha Omega Laboratories containing zinc chloride, chapparal leaf extract (*Larrea mexicata*), graviola leaf extract (*Annona muricata*), oleander leaf extract (*Nerium oleander*), Bloodroot (*S. canadensis*) and glycerine [276]. The companies’ internal cancer tonic Cansema (Amazon) Tonic III does not list bloodroot as an ingredient. There are no recorded clinical trials of black salve despite vendor claims of better than 98% skin cancer success rates [276] with minimal adverse reactions [276]. Some vendors suggest black salve can be used to treat melanoma [276,277].

There are significant concerns about the effectiveness of black salves. Cases of extensive tissue destruction [278] and recurrent disease resulting in death have been reported from its use [279]. Of the two histologically confirmed cases of melanoma treated with black salve, both developed metastatic disease [280,281].

Although vendors often list the ingredients of their black salve products, the concentration and shelf life of *S. canadensis* alkaloids is not stated and may not be known. To date, only one study has reported on the chemical composition of a single sample of black salve. This was a qualitative assessment that sought to detect constituents; their concentrations however were not reported [282]. *S. canadensis* alkaloids, as described, experience natural seasonal concentration variations and are impacted by a number of environmental and genetic factors. With compounds having broad bioactivity at micromolar concentrations, changes in salve alkaloid levels could result in unintended clinical effects.

## 7. Future Application and Risks of Using *S. canadensis*

The use of *S. canadensis* represents two different approaches to the therapeutic use of plants. Traditional medicines are developed by generations of indigenous people identifying and experimenting with biologically active natural products from very specific sources within their environment. These remedies often contain complex mixtures of organic chemicals derived from multiple botanical sources. In contrast, Western medicine has focused on identifying individual bioactive compounds with drug development potential via high throughput screening against molecular targets.

Both the traditional use and modern biological investigations of bloodroot suggest it warrants further study as a phytochemical extract and as a source of individual alkaloid lead compounds. Native Americans utilized the plant to treat a range of symptoms and conditions. Examining the in vitro bioactivity of individual alkaloids mechanistically supports these traditional uses. The anti-inflammatory, antimicrobial and anticancer capabilities of *S. Canadensis,* in particular, suggest further research is warranted.

Rational drug design has focused on a one compound, one target approach to minimize undesirable side effects that may arise from binding to secondary targets [283]. This philosophy has been challenged with the realization that drug promiscuity with polypharmacology is a mechanism by which a number of drugs exert their desired action [284]. Exquisitely selective compounds may reduce the risk of toxicity but may also less effectively treat disease. The alkaloids present in *S. canadensis* are phytoalexin allelochemicals capable of disrupting multiple cellular pathways. This broad bioactivity may exclude bloodroot alkaloids as classical drug candidates due to toxicity concerns. However, their ethnopharmacological history of use suggests the development of therapeutically effective doses, with toxicity levels acceptable to patients.

Indiscriminate cytotoxicity is of concern relating to black salve use. One in vitro study has suggested sanguinarine has a concentration dependent therapeutic window with a 2 μM concentration showing preferential cytotoxicity to the A431 squamous cancer cell line compared to normal keratinocytes, this discriminating ability being lost at a 5 µM concentration [285]. Other studies however have not supported this finding, with sanguinarine having equivalent cytotoxicity between tumor and normal cell lines [24] or actually having a greater cytotoxic effect on skin fibroblasts than the same A431 cell line [155]. Even if a sanguinarine therapeutic window exists, it is doubtful that natural therapies derived directly from *S. canadensis* rhizomes with their variable alkaloid composition would be able to target such a narrow pharmacological sweet spot. An assessment of black salve composition is urgently required to determine patient alkaloid exposures. This may offer an explanation for cases of skin cancer persistence due to subtherapeutic alkaloid levels and extensive tissue necrosis due to toxic alkaloid concentrations.

While the cellular cytotoxicity of individual alkaloids has been studied, the cytotoxicity of polyalkaloid extracts has received less attention. Antimicrobial research has shown *S. canadensis* alkaloid combinations exhibit four-fold greater potency than individual alkaloids against *H. pylori* [206] while alkaloid combinations have shown up to 10-fold improved killing of *Trypanosoma brucei* [286] suggesting they may have a synergistic effect. Whether phytochemical mixtures like black salve result in cellular cytotoxicity at concentrations lower than currently determined for individual alkaloids remains to be investigated.

The cytotoxic effect of an alkaloid mixture derived from *Chelidonium majus* has been assessed against murine fibroblast NIH/3T3, mouse melanoma B16F10, and human breast cancer MCF7 cell lines. The alkaloid mixture contained 3.3 μg/mL each of sanguinarine, protopine, allocryptopine, chelidonine and stylopine. It showed preferential melanoma cytotoxicity with 45% cell viability after 40 min compared to 75% normal cell viability. Unfortunately, a comparison with individual alkaloid cytotoxicity to determine the presence of alkaloid mixture synergy was not reported [287]. Although normal cells experienced a nearly two-fold greater alkaloid penetration, they experienced less cytotoxic effect due to preferential allocryptopine and stylopine permeability. In contrast, cancer cell lines showed greater penetration by sanguinarine and chelidonine.

The carcinogenic or mutagenic risk posed by *S. canadensis* botanical therapeutics is another area of clinical concern. *S. canadensis* mouthwash products cause leukoplakia, a pre-malignant condition. Skin cancers often develop within a field of genetically damaged cells [288], exposure to potentially genotoxic alkaloids could initiate or promote tumor development. Existing cancers if not destroyed by black salve may sustain further genetic damage rendering them more aggressive and treatment resistant.

Research specifically looking at sanguinarine’s carcinogenic potential has yielded contradictory results. Female Swiss albino mice exposed to initiator 1,3-dimethylbutylamine (DMBA) with sanguinarine (4.5 μM concentration) as a single application did not show an increased tumor development rate compared to DMBA application alone. In the same experiment, mice initiated with DMBA exposed to twice weekly topical 1.5 μM sanguinarine for 25 weeks developed an earlier onset of tumorigenesis and increase in the mean tumor number per mouse from 5 for the DMBA/TPA initiation group to 7.07 for the DMBA/TPA initiation followed by 25-week sanguinarine application group [289]. This result suggests sanguinarine can act as a tumor promoter.

Another study however, in female SKH-1 hairless mice, showed sanguinarine had a protective effect against UVB mediated damage suggesting it may have a role in the prevention of skin cancer. A topical application of 5 μmol sanguinarine, either as a pretreatment 30 min prior to UVB exposure, or a post-treatment 5 min after UVB exposure significantly reduced UVB induced skin oedema, hyperplasia and leukocyte infiltration [290]. Sanguinarine has been shown to induce apoptosis in UVB damaged HaCaT keratinocytes [291]. It also has an anti-oxidant effect achieved by blunting inflammatory cell oxidative bursts, not by operating as a free radical scavenger, but by disrupting NADPH oxidase [137]. By eliminating UV damaged cells and reducing UV-induced inflammatory changes, sanguinarine may protect against the development of skin cancer. The mixed story of cancer promotion and protection in different murine models requires clarification with future research especially considering these agents are currently being used by patients.

Patients believe natural plant therapies to be safer than manufactured pharmaceuticals [292]. Approximately 45% of the population in Western countries have used complementary and alternative medicine (CAM) within a 12-month period [293]. Concerns exist about the lack of regulatory control over the natural therapy sector and how this impacts quality control and patient safety [294]. With pharmaceutical products, manufacturers must provide regulators with safety and efficacy evidence before their product can be sold. In many countries, natural therapies may be sold without first demonstrating safety and efficacy, regulators bearing the burden of proving an agent is unsafe before it can be removed from the market [295]. A recent review of over the counter traditional Chinese medicine found that of 26 products, 92% had pharmaceutical, heavy metal contamination or substitution [296]. Safety is a key component of any human therapy; the lack of natural therapy regulatory control can leave patients vulnerable to toxicities that may be severe and life threatening.

## 8. Conclusions

*S. canadensis* is a traditional medicine with a variety of ethnopharmacological uses. Currently, the plant is used as an agricultural feed supplement; homeopathic remedy for menopausal flushing and osteoarthritis; and a cancer salve constituent. *S. canadensis* contains a number of bioactive alkaloids that have a range of molecular effects. Many factors have been shown to influence plant alkaloid concentrations which may impact the alkaloid composition of current bloodroot therapeutics.

*S. canadensis* cancer salves have a controversial history and have never been assessed in a clinical trial. The natural variation in *S. canadensis* alkaloids may offer an explanation for differing black salve clinical effects that warrants further exploration. The association of *S. canadensis* containing mouthwash with the development of leukoplakia and a murine model suggesting sanguinarine acts as a tumor promoter highlights the urgent need for studies into black salve carcinogenesis. Although regulators have issued warnings regarding black salve and have criminalized its importation in some jurisdictions, the therapy remains popular with patients interested in CAM.

Clinicians should engage with patients and discourage black salve use, especially for high risk skin cancers and those in cosmetically sensitive areas. As patients are currently using black salve, carefully designed observational studies could provide the fastest means of determining human in vivo toxicity and an assessment of its carcinogenic potential through tissue analysis of escharotic treated lesions. While such a reverse toxicology approach is not typical, removal of black salve treated areas could improve patient safety while providing relevant human tissue data. *S. canadensis* is a plant of therapeutic possibilities; the first step, however, should be to fully assess its safety.

## Figures and Tables

**Table 1:** Comparison of the cytotoxicity of major and minor QBA (IC_50_) [155,157].

Alkaloids (IC_50_, μg/mL)	HL-60	HeLa	KF-II	A431
Sanguilutine	0.04	0.46	–	–
Sanguirubine	0.12	0.68	0.22	0.7
Chelilutine	0.16	0.84	–	–
Chelirubine	0.1	0.37	0.2	0.28
Sanguinarine	0.34	0.7	0.5	0.7
Chelerythrine	0.17	0.48	0.58	1.44

**Table 2:** Anticancer Effects of *S. canadensis* alkaloids.

Molecular Target	Cellular Effect/Significance	Ref.
**Sanguinarine**		
Topoisomerase II	Prevents DNA break repair	[114]
Telomere Capping	Induces rapid apoptosis	[164]
Oncogenes C-myc, KRAS, C-kit	Expressed in various tumors	[120]
H-DNA	Haematological and colorectal tumor expression	[127]
Bcl-2 family	Apoptosis induction	[165]
ERKs	Apoptosis induction	[166]
NF-κB	Role in proliferation, migration, apoptosis	[162]
DR-5	TRAIL mediated apoptosis	[167]
Endoplasmic Reticulum	Unfolded Protein Response	[139]
VEGF-A	Impairs tumor neovascularization	[148]
Glutathione	Depletion amplifies oxidative stress	[24]
Anti-microtubule	Inhibits cell proliferation	[121]
**Chelerythrine**		
Bcl-XL and Bcl-2	Apoptosis Induction	[153]
Telomere Capping	Induces rapid apoptosis	[168]
Succinate	Cytochrome c release	[169]
NADH dehydrogenase	Apoptosome assembly	[169]
Glutaminase	Blocks Tumor Glutamine use for energy	[170]
mTOR	Overexpressed in Melanoma	[154]
Phospholipase D	Associated with angiogenesis/metastasis	[171]
MRCK	Impairs tumor migration	[172]
Tubulin Polymerization	Impairs cell division	[122]
MAPK	Activation results in apoptosis	[173]
**Minor QBAs**		
RIP1	Ripoptosome Formation	[158]
Unknown	Apoptosis ROS independent	[156]
Anti-microtubule	Impaired mitosis	[174]
**Protopin Alkaloids**		
Antioxidant effect	May antagonize the cytotoxic effects of other alkaloids	[175]
EGFR/ICAM-1	Reduced expression impairs metastasis	[161]
